# Cognitive Impairments Accompanying Rodent Mild Traumatic Brain Injury Involve p53-Dependent Neuronal Cell Death and Are Ameliorated by the Tetrahydrobenzothiazole PFT-α

**DOI:** 10.1371/journal.pone.0079837

**Published:** 2013-11-28

**Authors:** Lital Rachmany, David Tweedie, Vardit Rubovitch, Qian-Sheng Yu, Yazhou Li, Jia-Yi Wang, Chaim G. Pick, Nigel H. Greig

**Affiliations:** 1 Department of Anatomy and Anthropology, Sackler School of Medicine and Sagol School of Neuroscience, Tel Aviv University, Tel Aviv, Israel; 2 Drug Design & Development Section, Translational Gerontology Branch, Intramural Research Program, National Institute on Aging, National Institutes of Health, Baltimore, Maryland, United States of America; 3 Graduate Institute of Medical Sciences, College of Medicine, Taipei Medical University, Taipei, Taiwan; National Insttitute on Drug Abuse, United States of America

## Abstract

With parallels to concussive mild traumatic brain injury (mTBI) occurring in humans, anesthetized mice subjected to a single 30 g weight drop mTBI event to the right parietal cortex exhibited significant diffuse neuronal degeneration that was accompanied by delayed impairments in recognition and spatial memory. To elucidate the involvement of reversible p53-dependent apoptosis in this neuronal loss and associated cognitive deficits, mice were subjected to experimental mTBI followed by the systemic administration of the tetrahydrobenzothiazole p53 inactivator, PFT-α, or vehicle. Neuronal loss was quantified immunohistochemically at 72 hr. post-injury by the use of fluoro-Jade B and NeuN within the dentate gyrus on both sides of the brain, and recognition and spatial memory were assessed by novel object recognition and Y-maze paradigms at 7 and 30 days post injury. Systemic administration of a single dose of PFT-α 1 hr. post-injury significantly ameliorated both neuronal cell death and cognitive impairments, which were no different from sham control animals. Cellular studies on human SH-SY5Y cells and rat primary neurons challenged with glutamate excitotoxicity and H_2_O_2_ induced oxidative stress, confirmed the ability of PFT-α and a close analog to protect against these TBI associated mechanisms mediating neuronal loss. These studies suggest that p53-dependent apoptotic mechanisms underpin the neuronal and cognitive losses accompanying mTBI, and that these are potentially reversible by p53 inactivation.

## Introduction

Traumatic brain injury (TBI) represents an important and growing worldwide public health concern. It is a commonly occurring injury in victims of sports and motor vehicle accidents, especially for young men [1,2], and of falls in the elderly [3]. According to the CDC (Centers for Disease Control and prevention) some 1.7 million people suffer from TBI annually within the United States alone and, of these, almost 80% are considered as mild cases [4]. Victims of TBI suffer from a broad range of short- and long-term physical, cognitive, and emotional impairments consequent to their brain damage.

The adverse outcome that mild TBI (mTBI) patients most commonly suffer is the occurrence of neurobehavioral problems or post-concussion syndrome (PCS) [5-7]. This is characterized by cognitive symptoms that include difficulties in concentrating, memory loss, a decreased speed of information processing, an inability to multitask, and difficulties in initiating and planning [7]. Previous research in a non-invasive closed head mTBI mouse model demonstrates that it induces cognitive and behavioral short- and long- term deficits [8-13] that, to a degree and similar to a number of other rodent models [14], mimic the human condition. Primary brain injury is induced by the immediate insult to the head, while the development of secondary brain injury takes place from minutes to days following the trauma [15]. Most of the damage apparent in mild injury patients derives from the secondary events of the trauma, which includes brain edema, inflammatory responses, free radical generation, glutamate-induced excito-toxicity and DNA damage [16-18]. When cellular damage is sufficiently profound, the pro-apoptotic protein, p53 will initiate the process of apoptosis. 

It is becoming increasingly evident that neuronal cell death may contribute to the cognitive deficits that appear following a TBI event [19]. Previous research from our laboratory has revealed the occurrence of diffuse neuronal cell death throughout the brain [20] together with elevated levels of p53 following mTBI in mice involving as little as 15 to 30 g impact [21]. The elegant work of others has, likewise, demonstrated elevations in p53 mRNA as well as protein levels within the hippocampus and cortex as a result of TBI [22-24]. TBI has additionally been described to induce the phosphorylation of p53 within the hippocampus [25]; thus increasing its stabilization and capacity to resist degradation pathways to, thereby, promote its ability to initiate apoptosis [26,27]. Elevations in p53 have been reported in the penumbra surrounding the core of both a stroke [28,29] and lesion induced by open head cortical impact injury [23], where its heightened levels correlated to the secondary contusion expansion [23]. The inactivation of the p53 signaling pathway resulted in a reduction in the volume of this secondary contusion and an improved outcome in both conditions [23,28,29], supporting a primary role of p53 in the neuronal cell dysfunction and death occurring around ischemia- and TBI-induced lesions.

The tetrahydrobenzothiazole analogue pifithrin alpha (PFT-α) is a synthetic agent that limits apoptosis through inhibition of p53-mediated transcription [30,31]. PFT-α has been reported to be beneficial across a wide array of neurodegenerative models, including ones relevant to schemic injury and stroke [28,29,32], ALS [33], Huntington’s disease [34] and Parkinson’s disease [35]. 

In light of the favorable activity of PFT-α across such a range of cellular and animal models involving neuronal dysfunction and loss, and the beneficial effect of the agent in reducing secondary lesion expansion following cortical impact injury [23], the focus of this study was to evaluate whether acute administration of PFT-α could ameliorate cognitive deficits resulting from mTBI, where apoptosis is diffuse [20] and a primary lesion is absent [11]. As TBI is associated with a massive release of excitatory amino acid neurotransmitters, particularly glutamate [36] whose extracellular availability detrimentally impacts neurons and astrocytes and results in over-stimulation of ionotropic and metabotropic glutamate receptors inducing successive Ca^2+^, Na^+^, and K^+^-fluxes [37-39], the ability of PFT-α to protect neuronal cultures from glutamate excitotoxicity, oxidative stress and natural degeneration was evaluated. Finally, to verify that the neuroprotection of PFT-α was due to its reported anti-apoptotic actions, brain slices from treated and untreated mTBI animals were immunostained with antibodies to allow visualization of degenerating (Fluoro-Jade B) and mature neurons (anti-NeuN).

## Materials and Methods

### Ethics Statement

The Ethics Committee of the Sackler Faculty of Medicine approved the experimental protocols (M-12-063), in compliance with the guidelines for animal experimentation of the National Research Council (Committee for the Update of the Guide for the Care and Use of Laboratory Animals, 2011). A minimal number of mice were used for the study and all efforts were made to minimize potential suffering.

### Pifithrin-α (PFT-α)

**Figure 1:**
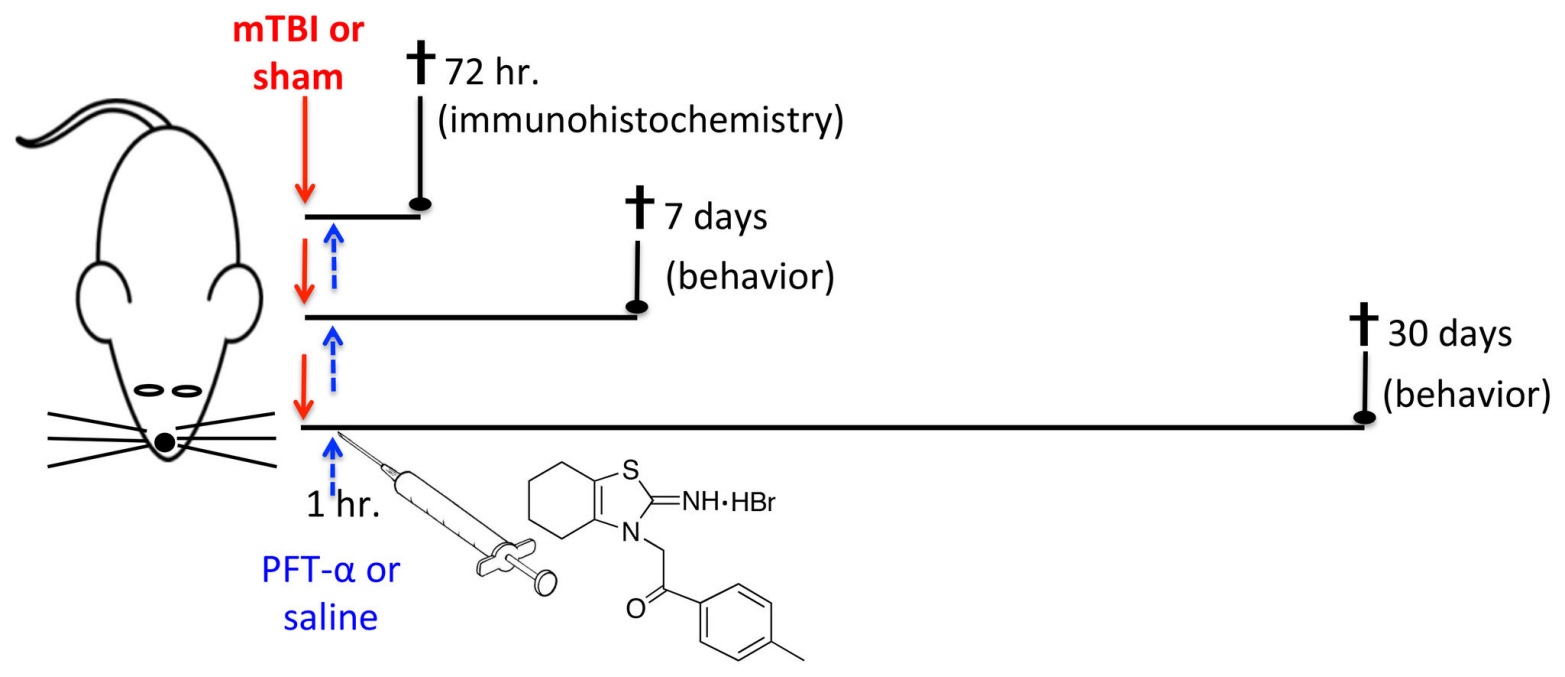
Time line of mouse studies. Anesthetized male ICR mice were subjected to either mTBI (a single 30 g weight drop) or a sham procedure (without weight drop) and 1 hr. later were administered either PFT-α (2 mg/kg, i.p.) or vehicle (0.2% DMSO/saline mixture, i.p.). Three parallel series of animals were then maintained for (i) 72 hr. and were prepared for immunohistochemical analyses of their brain tissue for quantification of degenerating neurons assessed by FJB and NeuN, (ii) 7 days and were behaviorally evaluated by novel object recognition and Y-maze paradigms, and (iii) 30 days and underwent similar behavior evaluation. The structure of PFT-α is shown as its synthesized HBr salt.

PFT-α [1-(4-methyl-phenyl)-2-(4,5,6,7-tetrahydro-2-imino-3(2H) benzothiazolyl)ethanone) was synthesized as its HBr salt, according to the route of Zhu et al. [31], (Figure **1**), and the close analog, Y-6-159, was likewise generated to confirm that activity was retained across p53 inactivators. Chemical characterization confirmed the structures of the desired compounds in high purity (>99%), which were later dissolved in 100% dimethyl sulfoxide (DMSO) for cell culture studies.

### Neuronal Cultures

Primary cultures: primary cortical cultures were isolated from E15 embryos obtained from timed-pregnant Sprague–Dawley rats, as described previously [40] and in accord with approved procedures by the NIH Animal Care and Use Committee. Specifically, dissected brain cortices from E15 embryos were pooled and digested for 20 min in pre-warmed (37°C) 1 ml/embryo of 0.05% trypsin–EDTA (0.2% (Invitrogen, La Jolla, CA)). Cortices were then triturated and diluted into plating media to approximately 2 ml per embryo. This plating media comprised Neurobasal media (Invitrogen), 2% heat-inactivated fetal bovine serum (Sigma-Aldrich, Milwaukee, WI), 2% B27 supplement (Invitrogen), 200 mM L-glutamine and 25 mM L-glutamate. Cell viability was evaluated by trypan blue staining (Invitrogen) and cells were plated at 3 × 10^4^ viable cells/well in 0.2 ml plating media into 96 well plates coated with 0.15–0.2% polyethyleneimine in 150 mM sodium borate, pH 8.5 (Sigma-Aldrich). Thereafter, plated cells were maintained in a humidified incubator (37 °C, 5% CO_2_, 95% air) and fed by 50% media exchange starting on the 4th day in vitro (DIV4) with feed media (plating media without serum or glutamate), with additional feedings thereafter.

To evaluate p53-dependent neuroprotection afforded by PFT-α, neuronal cultures were either permitted to naturally degenerate or were challenged to glutamate (Sigma-Aldrich) excess (DIV7 and DIV8). This glutamate dose (100 µM) was selected from preliminary time- and dose-response studies, sufficient to induce mild cellular dysfunction and loss. Cultures were pre-treated for 1 hr. with PFT-α (diluted to less than 0.5% DMSO) and challenged with glutamate followed by the assessment of cell viability 24 hr. To evaluate protection against natural degeneration, PFT-α was added to their media over 24 hr. Cellular viability was quantified by MTS assay using the CellTiter 96 Aqueous One Solution Cell Proliferation Assay kit (Promega, Madison, WI, USA) according to the manufacturer’s instructions.

Human SH-SY5Y cultures: SH-SY5Y cells from American Type Culture Collection (ATCC, Manassa, VA, USA) were cultured (1:1 mixture) in Eagle’s minimum essential medium and Ham’s F12 medium containing fetal bovine serum (10%) and penicillin-streptomycin (1%) (Invitrogen), and were plated at a density of 20,000 cells/100 µl in 96-well plates (37 °C in a humidified incubator with 5 % CO_2_ and 95 % air). After 24 hr., cells were exposed to PFT-α analog Y-6-159 (1–10 µM) for 60 min and then were challenged with glutamate (100 mM) or oxidative stress (H_2_O_2_: 500 μM) for 24 hr. These glutamate and H_2_O_2_ concentrations were selected from initial dose–response studies to provoke a significant but incomplete level of cellular toxicity. Thereafter, cellular viability was quantified by MTS assay (Promega).

### Animal Studies

Male ICR mice weighing 30–40 g were kept five per cage under a constant 12-hr. light/dark cycle, at room temperature (22±2°C). Food (Purina rodent chow) and water were available *ad libitum*. Each mouse was used for one experiment and for one time point alone. 

### Mild traumatic brain injury (mTBI)

Experimental mTBI was induced using the concussive closed scalp, head trauma device described previously [9,13]. Mice were anesthetized by inhalation of Isoflurane in a closed glass chamber and placed under a metal tube device where the opening was positioned directly over the animal’s head just anterior to the right ear. The animals were held in such a way that the force of impact to the skull generated anterio-lateral movements without any rotational movements, analogous to those that occur during closed head injury in car accidents. The injury was induced by dropping a blunted cylindrical metal weight (30 g), inside the metal tube device (inner diameter 13 mm) from a height of 80 cm. Mice were placed back in their cages to allow for recovery from the anesthesia and TBI, immediately after the induction of the injury. The potential effects of the weight drop injury were studied at 72 hr. and at 7 and 30 days following the trauma (Figure **1**). For each time point, different groups of mice were utilized with a minimum of 9 mice per group.

### PFT-α administration

Animals were administered PFT-α 2 mg/kg body weight by the intraperitoneal (i.p.) route 1 hr. post injury, drug vehicle control animals were treated with a 0.2% DMSO/saline mixture. PFT-α was maintained in 100% DMSO as a concentrated stock and diluted in saline immediately prior to administration.

### Behavioral tests

Behavioral analyses were undertaken 7 and 30 days after the animals received the mTBI injury (Figure **1**). The effects of mTBI on mouse cognition were assessed using the following behavioral paradigms: the novel object recognition (NOR) and the Y maze. The assessments were conducted once per day at approximately the same time each day during the light phase of the cycle.

### Novel object recognition paradigm

The NOR task was used to evaluate recognition memory in mice as previously described [10,12]. This task is based on the innate tendency of rodents to explore new objects within their environment. The use of this natural tendency allows one to determine whether a mouse can discriminate between a familiar and a novel object. Mice were individually habituated to an open field Plexiglass arena (59 x 59 x 20 cm) for a period of 5 min. Twenty-four hours later, in the acquisition phase, two identical objects (A and B) were placed in a symmetrical position within the arena. The objects were sufficiently large to ensure that the mice could neither move nor climb over them. During the memory recognition assessment phase that was assessed 24 hr. thereafter, one of the objects (A or B) was randomly replaced by a novel one (C), and the mouse exploratory behavior was analyzed over a 5 min period. Exploration of an object was defined as rearing on the object, sniffing it at a distance of less than 2 cm and/or touching it with the nose. Successful recognition was represented by preferential exploration of the novel object over the familiar object. The time spent by each mouse exploring the novel object over the familiar object was recorded and used to generate a preference index as initially described by Dix and Aggleton [41]. A discrimination preference index was calculated as following: (time spent near the new object minus time spent near the old object) / (time spent near the new object plus time spent near the old object). After each session, the objects and arena were thoroughly cleaned with 70% ethanol to prevent odor recognition.

### Y maze paradigm

The Y maze task assesses rodent spatial memory; it is based upon observing the preference of the animal for a ‘new’ location over a ‘familiar’ location on two separate occasions. The maze is composed of three black Plexiglass arms separated by a 120° angle at a central axis [42]. Each arm was identical in construction (8 x 30 x 15 cm) yet distinctive by the presence of different visual cues placed at the ends of the arms (i.e. a triangle, square, or a circle). For each animal one arm was randomly selected as the “start” arm, during the first trial (5 min in duration) one of the two remaining arms was randomly blocked whereas on the second trial (2 min in duration) all arms of the maze were open. The two behavioral trials were separated by a 2 min interval during which the mouse was returned to its home cage. The time each mouse spent by in the arms was recorded and used to generate a preference index as initially described by Dix and Aggleton [41]. The discrimination preference index was calculated as follows: (time spent in new arm minus time spent in familiar arm) / (time spent in new arm plus time spent in familiar arm). Between the trials and animals, the maze was cleaned using a 70% ethanol solution and dried in order to prevent any olfactory recognition. 

### Immunohistochemistry Staining

To assess for early changes neuronal cell viability, at 72 hr. post mTBI and sections were stained for NeuN (a marker of mature neurons) and Fluoro-Jade B (FJB: a marker of degenerating neurons). A ratio of the numbers of degenerating neurons over the number of mature neurons was used as an index of trauma-induced cellular health. Mice were anaesthetized with a combination of ketamine (100 mg/kg) and xylazine (10 mg/kg) and perfused transcardially with 10 ml of phosphate buffered saline (PBS) followed by perfusion with 20 ml of a 4% paraformaldehyde (PFA) buffer. The brains were post-fixed overnight in the same fixative solution and then transferred to 1% PFA. The brains were submerged in a 30% sucrose solution for 48 hr. prior to sectioning. Thirty micrometer thick free floating coronal sections were prepared on a cryostat. The sections were collected in a cryoprotectant solution containing phosphate buffer, ethylene glycol, and glycerin, and stored at -20°C. Every twelfth section throughout the brain was stained with a mouse primary antibody that detects NeuN (Millipore; MAB377, diluted 1 in 50 in incubation buffer), after the incubation with primary antibody the sections were washed and incubated with a Cy3 labeled anti-mouse secondary antibody (Jackson; 715-165-150, diluted 1 in 300 incubation buffer). The probed sections were mounted onto 2% gelatin coated slides and stained with FJB (Millipore; AG310) as described by Schmued and Hopkins [43]. In light of the diffuse, rather than local, cellular dysfunction and loss that has been described to occur across both cerebral hemispheres in our mTBI model [20,21], both the ipsi- and contra-lateral hippocampi were analyzed and pooled together to attain stricter statistical analysis. The means of 6 to 10 mouse brains per treatment were used to assess the FJB/NeuN ratios for each treatment group. The slides were observed using a Zeiss Axiovert 200 fluorescence microscope (Zeiss).

### Data analysis

All results are shown as mean ± standard error of mean values. Data from primary cell culture studies were subjected to one-way analysis of variance (ANOVA) and Dunnett’s multiple comparison t-test. Data from animal studies were analyzed using SPSS 17 software (Genius Systems, Petah Tikva, Israel). A one way ANOVA was used to analyze data for the behavioral paradigms: NOR and Y maze, the p values of post hoc tests were adjusted using Bonferroni or Fisher’s least significant difference (LSD) test utilizing a nominal significance level of 0.05. 

## Results

### Neuronal Cultures

#### The effects of PFT-α on glutamate/oxidative stress toxicity across neuronal cultures

**Figure 2:**
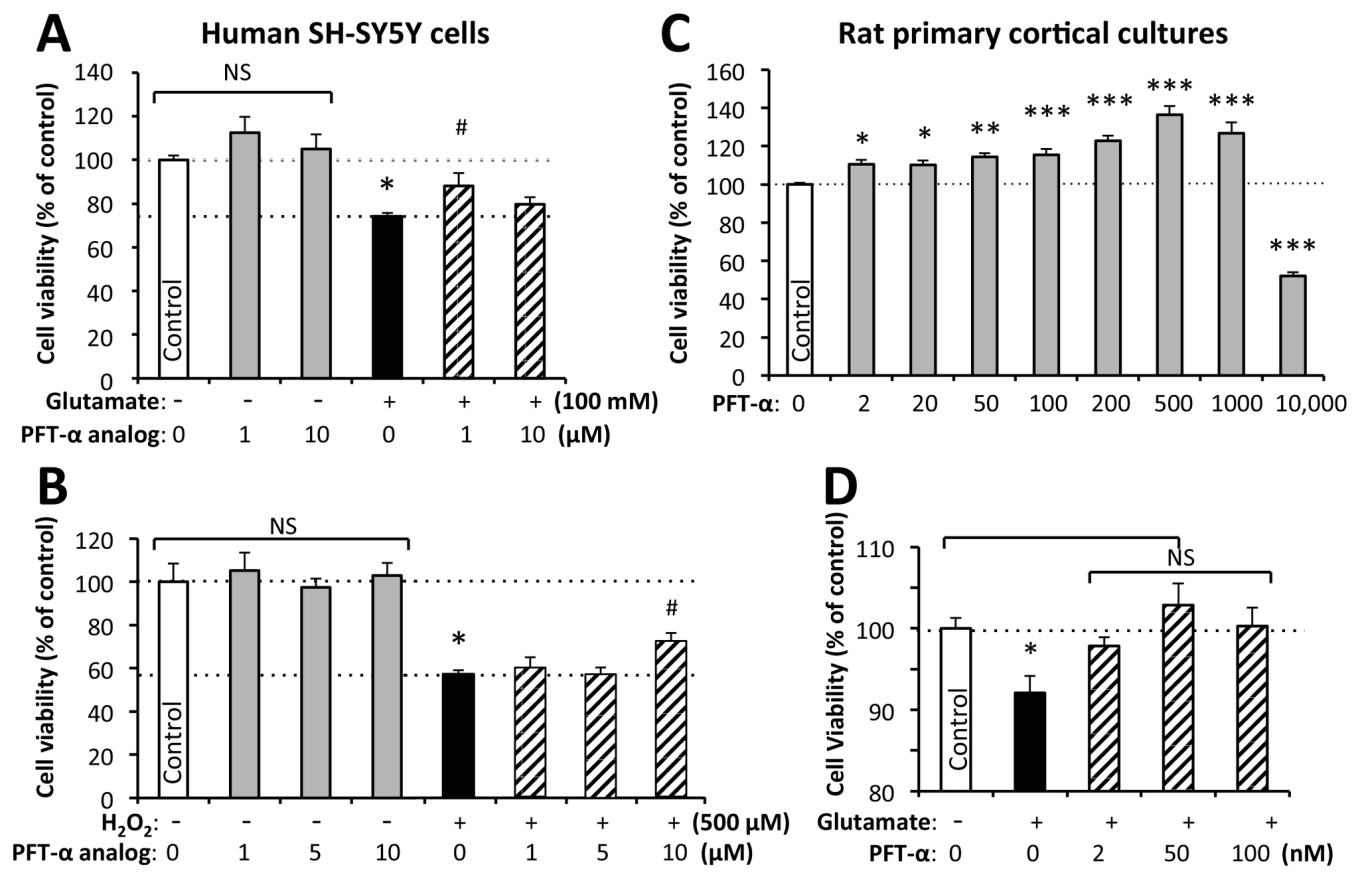
p53 inhibition by PFT-α/analog inhibits glutamate-induced excitotoxicity and oxidative stress mediated loss of cell viability in neuronal cultures. Human SH-SY5Y cells were subjected to p53 inactivation (PFT-α analog 1 to 10 μM) and then challenged with (**A**) glutamate (100 mM) excitotoxicity or (**B**) oxidative stress (H_2_O_2_: 500 μM). These insults alone significantly reduced cellular viability (* p<0.05 vs. control, Dunnetts t-test), which was mitigated by p53 inactivation (# p<0.05 vs. glutamate alone, Dunnetts t-test). (**C**) Rat primary cortical neuron cultures undergo time-dependent degeneration [44] that was mitigated by the addition of PFT-α (2 nM to 1 μM; * p<0.05, ** p<0.01, *** p<0.001 vs. untreated controls that are expressed as 100% (Dunnett’s t-test). A 10 μM PFT-α concentration proved to be toxic to primary neurons (*** p<0.001 vs. untreated controls; Dunnett’s t-test). (**D**) In an alike manner to SH-SY5Y cells, exposure of primary cortical neurons to glutamate (100 μM) resulted in reduced survival (* p<0.05 vs. control, Dunnetts t-test),) and pre-treatment with 2 to 100 nM PFT-α ameliorated this (NS not significantly different from untreated controls, Dunnetts t-test). Analysis of viable neurons was undertaken by MTS assay at 24 hr.

As glutamate-induced excitotoxicity and oxidative stress together with natural degeneration [44], are considered to underpin, in part, the cellular loss in brain after a TBI insult [19,23,29], neuronal cultures were pretreated with vehicle or PFT-α/analog (primary cortical cells: 0, 2 nM to 10 μM PFT-α; human SH-SY5Y cells 1 to 10 μM PFT-α analog Y-6-159) and, 1 hr. later were exposed to glutamate (SH-SY5Y cells: 100 mM; primary cells: 100 μM, for 24 hr.) or oxidative stress (H_2_O_2_: 500 μM, 24 hr.) or no insult for naturally degenerating primary cortical cultures. The percent of neuronal survivals at 24 hr. are shown in Figure **2**. 

In line with prior studies [10], human SH-SY5Y cells proved susceptible to glutamate-mediated excitoxicity that induced a ~26% cellular loss (*p<0.05 vs. control), which was mitigated by p53 inhibition (Figure **2A**). Likewise, challenge with oxidative stress induced a ~43% loss of cell viability (*p<0.05 vs. control) that was similarly ameliorated by p53 inhibition (Figure **2B**). In accord with former studies [44], primary neurons undergo continuous time-dependent degeneration when maintained in culture (Figure **2C**), which was mitigated by PFT-α on comparison of treated with untreated control cultures. Likewise, primary cortical neurons proved vulnerable to glutamate, which induced a mild cellular loss (~10% loss vs. control *p<0.05 vs. controls) that was mitigated by PFT-α 2-100 nM concentrations (Figure **2D**). In general, PFT-α was well tolerated by SH-SY5Y and primary neuron cultures, except at concentrations approaching 10 μM for the latter. 

### Animal Studies

#### The effects of PFT-α treatment after injury on the NOR paradigm

**Figure 3:**
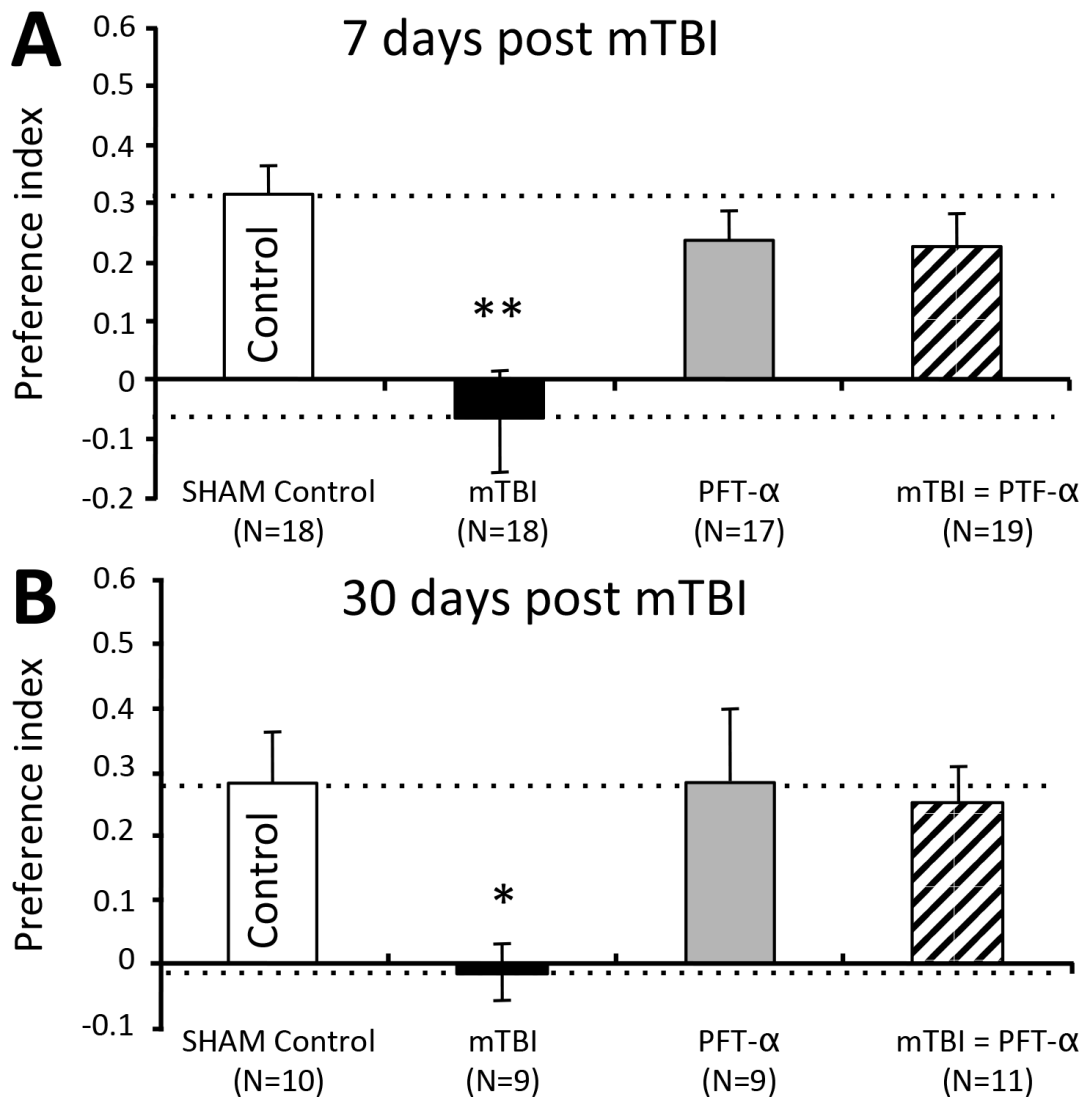
PFT-α inhibits mTBI-induce deficits in novel object recognition. (**A**) PFT-α administration 1 hr. post trauma ameliorated mTBI visual memory deficits. mTBI mice had a significantly lower visual memory compared with all other groups, a deficit that was reversed with the administration of PFT-α both 7 days post trauma (**p<0.01; Bonferroni *post* *hoc* [F_(3,68)_= 7.388, *p*<0.001]), and (**B**) 30 days post trauma (*p<0.05; Fisher’s LSD *post* *hoc* [F_(3,38)_= 3.104, *p*=0.039]). Performance of mice was quantitatively assessed as a preference index, calculated as (time near the new object - time near the old object)/(time near the new object + time near the old object). Values are mean ± SEM, of n= 9 - 19.

The NOR was used in order to examine the visual recognition memory of the mice at defined time points after injury. Seven days after the mTBI event the vehicle treated, injured mice exhibited impairments in visual memory when compared with all other treatment groups. However, the mTBI mice treated with PFT-α 1hr. post injury demonstrated a complete recovery of the loss of visual memory. One-way ANOVA revealed a significant effect of group [F_(3,68)_= 7.388, *p*<0.001]. Bonferroni *post hoc* analysis revealed that the preference index of the mTBI mice was significantly lower than the other groups (**p<0.01, Figure **3A**). Parallel groups of different mice were similarly evaluated 30 days after injury to characterize the long-term influence of PFT-α on the NOR assessment. Even at 30 days following injury, the vehicle mTBI animals demonstrated impairments in object recognition when compared with the other treatment groups (Figure **3B**). In contrast, PFT-α treated mTBI mice (1 hr. post injury) presented with no impairments in visual memory. One-way ANOVA revealed a significant effect of group [F_(3,38)_= 3.104, *p*=0.039]. Fisher’s LSD *post hoc* analysis revealed that the preference index of the mTBI mice was significantly lower compared with all other groups (*p<0.05, Figure **3A**). At both time points studied, sham PFT-α mice (no mTBI) demonstrated no differences in NOR performance compared to that of sham control mice.

#### The effects of PFT-α treatment after injury on the Y maze paradigm

**Figure 4:**
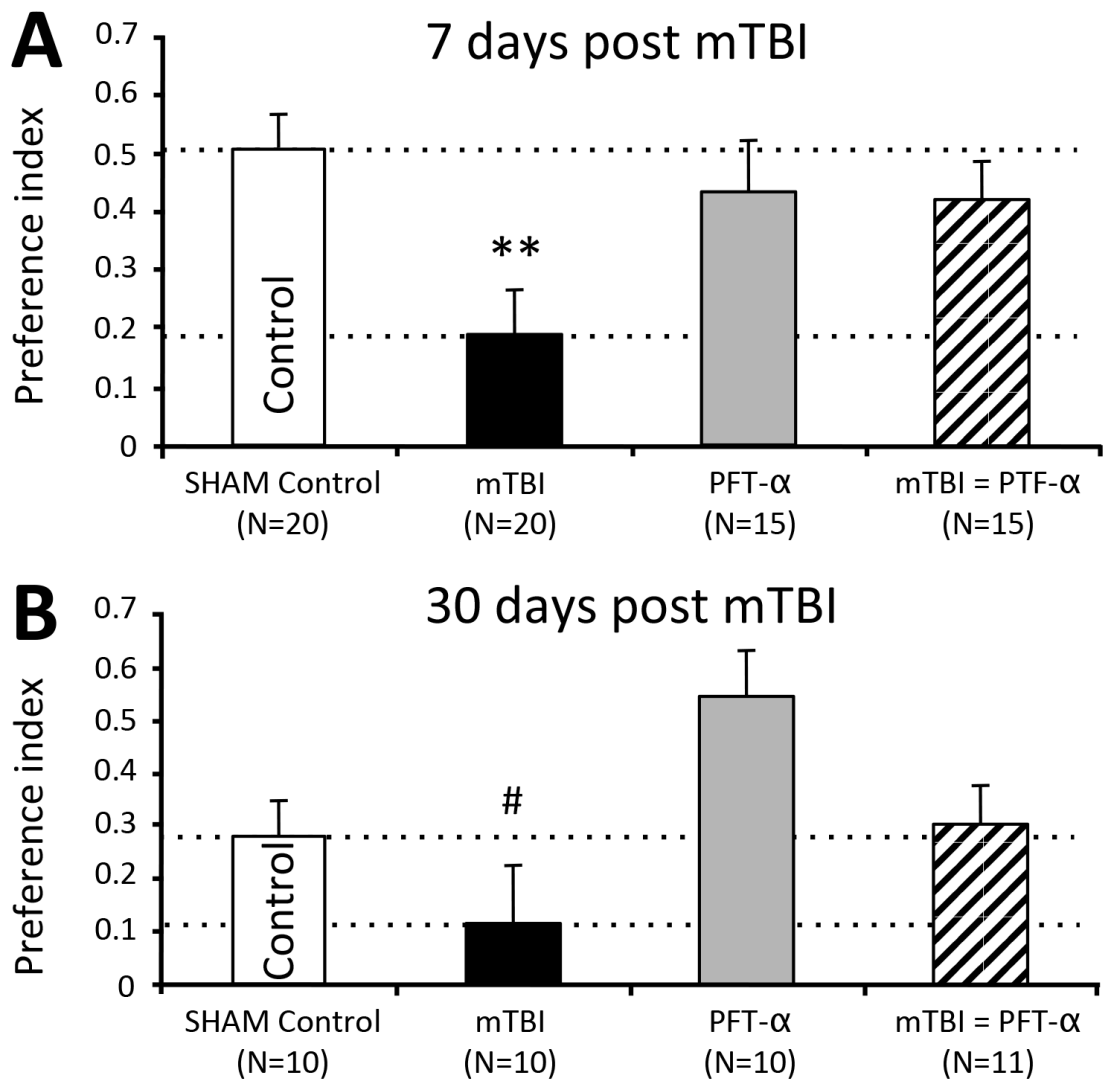
PFT-α inhibits mTBI-induce deficits in Y maze. (**A**) PFT-α administration 1 hr. post trauma improved mTBI spatial memory deficits. mTBI mice had a significantly lower visual memory compared with all other groups, a deficit that was corrected with the administration of PFT-α 7 days post trauma (**p<0.01; Fisher’s LSD *post* *hoc* [F_(3,72)_=4.155, *p*=0.009]). (**B**) 30 days post trauma the differences between mTBI and PFT-α mice reached statistical significance (#p<0.01; Bonferroni *post* *hoc* [F_(3,45)_= 4.337, *p*=0.009]). Performance of mice was quantitatively assessed as a preference index, calculated as (time at the new arm - time at the old arm)/(time at the new arm + time at the old arm). Values are mean ± SEM, of n= 10 - 20.

The Y maze paradigm was used to examine the spatial memory of the animals. Y maze paradigm measurements obtained from mice at 7 days post trauma, indicated that there was a significant difference between the treatment groups. One-way ANOVA revealed a significant change [F_(3,72)_=4.155, *p*=0.009], a Fisher’s LSD *post hoc* analysis revealed that the vehicle treated mTBI mice had significantly lower spatial memory abilities when compared with the other treatment groups (**p<0.01, Figure **4A**). At 30 days after injury one-way ANOVA revealed a significant difference between groups [F_(3,45)_= 4.337, *p*=0.009; Figure **4B**]. Likewise, mTBI challenged mice demonstrated lower spatial memory skills compared to other groups. Bonferroni *post hoc* analysis revealed that the spatial memory capabilities of the vehicle treated mTBI mice were impaired when compared to the PFT-α sham mice (PFT-α, no mTBI, #p<0.01, Figure **4B**).

#### The effects of PFT-α on mTBI-induced degenerating neurons in the dentate gyrus

**Figure 5:**
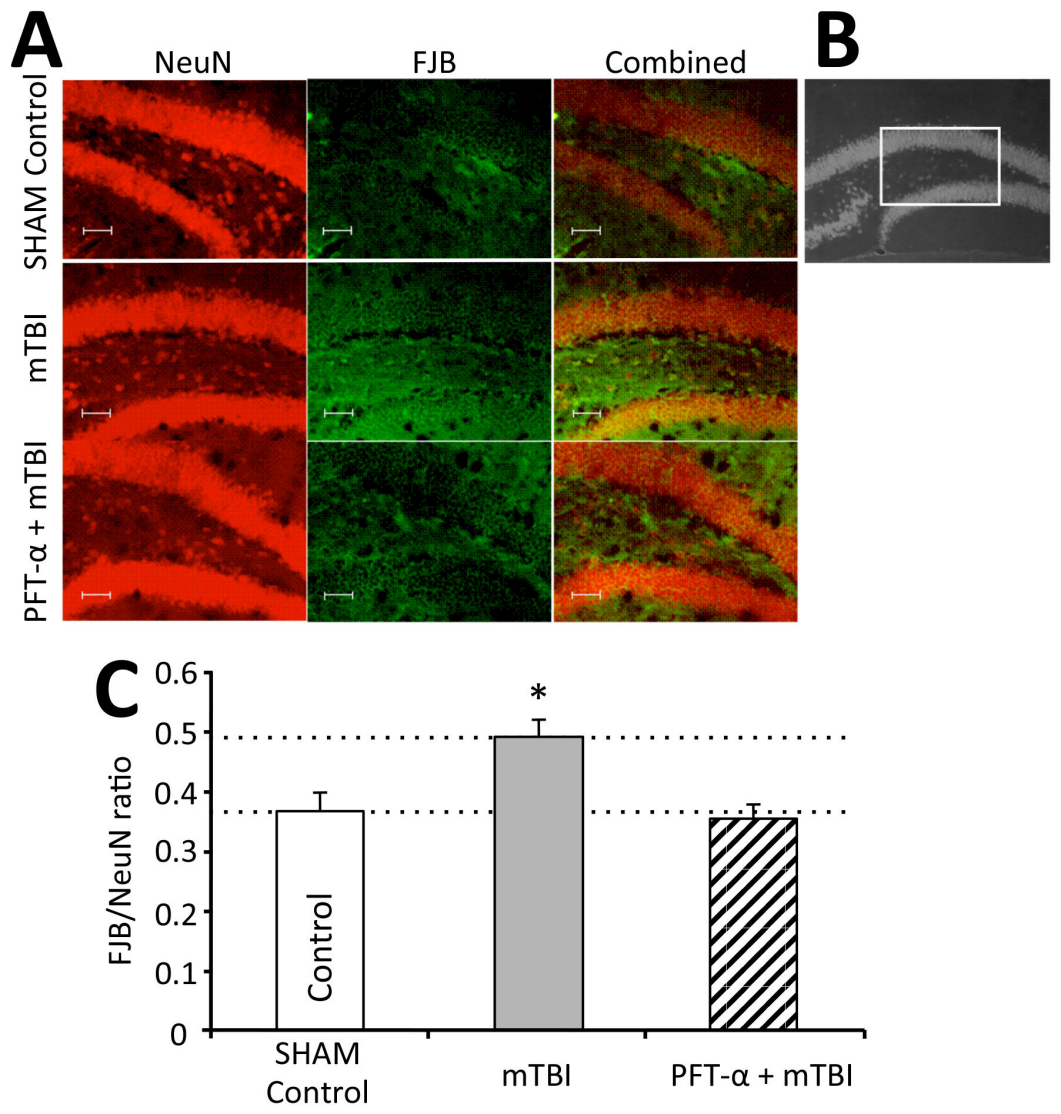
PFT-α mitigates mTBI-induced degeneration of neurons in the dentate gyrus. (**A**) Representative images of Fluoro Jade B (FJB) (green) and NeuN (red) positive neurons in the dentate gyrus 72 hr. after mTBI. Scale bar= 100µm. (**B**) The field in the box indicates the hilus of the dentate gyrus, which is represented in a higher magnification. (**C**) Bar graph shows the quantification of neuronal degeneration in the dentate gyrus as a ratio of number of neurons positively stained with FJB (degenerating neurons) divided by neurons positively stained with anti-NeuN in sham control, mTBI and mTBI PFT-α groups. (**P<0.01; Bonferroni *post* *hoc* [F_(2,19)_= 9.219, *p*=0.002). Values are mean ± SEM, of n= 6 - 10 mouse brains.

Mouse brain sections were prepared from animals 72 hr. after injury. Representative sections are shown in Figure **5A/B**, that were double-stained with FJB (shown in green) and antibodies selective for NeuN (shown in red). The FJB/NeuN ratio was calculated (the number of neurons undergoing degeneration (FJB stained) divided by the number of mature neurons (anti-NeuN stained). When numbers of mature neurons remain unchanged, as occurred in our study (there were no statistical differences in the mature neuron number between the groups: sham, mTBI, and mTBI+PFT-α mean values were 43.6, 34.1 and 46.2 respectively; p (ANOVA)=0.105)), a rise in the FJB/NeuN ratio occurs in the presence of an increased number of degenerating neurons.

The ratio was thereby used in order to define the fraction of degenerating neurons compared with the total number of neurons in the observed field within the hilus of the dentate gyrus. One-way ANOVA revealed a significant difference between groups [F_(2,17)_= 8.228, *p*=0.004]. A Bonferroni *post hoc* analysis showed a significant increase in the FJB/NeuN ratio in the mTBI alone compared with the sham mice. In addition, in mice subjected to mTBI and treated with PFT-α, the FJB/NeuN ratio was significantly lower versus the mTBI alone mice (0.358±0.02 and 0.495±0.06 in mTBI+ PFT-α and mTBI mice, respectively). No differences were found between the mTBI PFT-α and the sham control group (0.369±0.07; N=6-10; **p<0.01) (Figure **5C**).

## Discussion

Our study supports a primary role for p53 in the delayed neuronal death that occurs following a mTBI incident. Our prior studies demonstrated that increased severity of impact to the head positively correlates with a rise in the number of pyknotic (where condensation and a reduction in the size of the cell and particularly its nucleus occur, associated with hyperchromatosis and indicative of advancement towards necrotic cell death) and apoptotic neurons throughout the cerebral cortex and hippocampus both ipsi- and contralateral to a closed head mTBI injury [21]. In these former studies, the pro- and anti-apoptotic markers p53 and Bcl2, respectively, were up regulated by as little as a 5 g insult when evaluated at 72 h [21], with degeneration of neurons and their processes occurring at insults of 15 g within cortical regions and 25 to 30 g in hippocampus and dentate gyrus, as assessed by silver staining with verification of apoptosis by TUNEL staining [20]. Herein, we confirm the occurrence of diffuse neuronal cell death by the use of FJB, an anionic fluorescein derivative that is widely used to label degenerating neurons ex-vivo within the brain [43] with quantification of total neuron numbers by the use of NeuN immunostaining [45]. Furthermore, we link this p53-dependent neuronal cell death to later cognitive impairment (assessed at 7 and 30 days) by the use of a p53 inactivator, PFT-α, through its mitigation of both.

Of the 1.7 million Americans that experience TBI annually by far the majority (over 80%) suffer mTBI [4] and endure a spectrum of short- and long-term neurological deficits, particularly cognitive impairments [46-48] that may appreciably impact quality of life even following recovery from any physical disabilities [49]. Albeit no single animal TBI model perfectly reproduces the human condition [14], our non-invasive weight drop mTBI model has well characterized features pertinent to human head injury that can transpire from a traffic accident, a sports injury, or a fall. In accord with other weight drop rodent models [50], diffuse axonal injury arises throughout the brain that leads to diffuse neuronal loss, neuroinflammation, and later cognitive impairments [9,11,20,21,51-53] that are prime features occurring in most human mTBI cases [18]. In contrast to open head and focal TBI animal models [14,54,55] in which a contusion or focal lesion ensues, an attribute associated with more serious TBI in humans, neither presents in our mTBI model [11].

In an open head controlled cortical impact (CCI) model of TBI in mice that results in a cerebral contusion, p53 levels were found elevated in brain within 15 min of TBI, further rose at 3, 6 and 12 h and were sustained for at least 24 hr., as assessed at the rim and center of the contusion [23]. This rapid rise in p53 protein levels preceded neuronal cell death and correlated well with the secondary expansion of the contusion volume. Interestingly, however, p53 levels in brain regions unassociated with CCI (i.e., contralaterally) were described as not different from sham animals [23]. The administration of the p53 inactivator PFT-α (6 or 8 mg/kg, i.p.) either prior to or, optimally, up to 3 h post CCI, substantially mitigated the rise in p53 expression and reduced the secondary brain tissue loss after trauma. This effect largely mirrors prior studies evaluating p53-induced apoptosis in cerebral ischemia [28,31] in which the infarct volume time-dependently increased with p53 expression [28,56]. Likewise, PFT-α (2 mg/kg, i.p.) mitigated the rise in p53 levels, reduced the infarct size and improved behavioral outcome with a therapeutic window of approximately 3 h [28], with higher (4.0 mg/kg) and lower (0.2 mg/kg) doses proving less effective. 

The current study hence followed our prior ones and used a 2 mg/kg PFT-α dose within the previously established therapeutic window to evaluate the role of p53 in the diffuse neuronal cell death that occurs in our concussive mTBI model. Furthermore, neuronal degeneration was assessed at 72 hr. post mTBI immunohistochemically using FJB in combination with NeuN to define neurons, in accord with prior studies defining the time of peak neuronal apoptosis following a TBI event [11,57]. Staining with Fluoro Jade B correlates relatively well with Tunnel staining [58], and has been utilized to demonstrate neurodegeneration in multiple models of TBI with varying levels of injury severity. Marked positive staining of neurons has been observed in the fluid percussion [59-61]; controlled cortical impact [62,63] and the open head weight drop injury model [64]. None of these models can be considered mild, as compared to our closed head weight drop model, and thus the findings of these studies differ somewhat from ours, in terms of the levels of positively stained neurons. As indicated by Hellmich and colleagues [61], the level of FJB staining is dependent on the level of injury severity. Interestingly, similar to our study, these investigators also observed a low level of FJB staining in sham animals; the biological relevance of this is unclear and would require additional studies to address the significance of FJB staining in control animals. 

Our behavioral evaluation post mTBI was undertaken at 7 and 30 days, in accord with previous time-dependent studies [10,52]. These involved (i) recognition memory and, (ii), spatial memory, which as in prior studies [8,10,12,42,51,52,65,66] were impaired by mTBI. The former refers to the ability to discriminate a previously encountered (familiar) item from a novel one; a task that has become a valuable tool in basic and preclinical research for investigating the neural basis of memory [67], and that has parallels to visual paired-comparison tasks in studies in humans and monkeys. Damage to the hippocampus is sufficient to produce impaired recognition memory [68-70]. The latter, spontaneous spatial memory in the Y-maze, is likewise considered a hippocampal-dependent test [70,71] and, importantly, both recognition and spatial memory are impaired in humans with mTBI [72]. The hippocampus, in particular, appears to be vulnerable to mTBI induced neuronal degeneration [20,21,73,74]. PFT-α readily enters the brain [31,35] and fully ameliorated both the neuronal loss (Figure **5**) and cognitive impairments (Figures **3** and **4**), thereby implicating p53 as an important mediator in mechanisms underpinning these. 

To further establish a role of p53 in mTBI associated neuronal dysfunction, we challenged cultured neural cells (human SH-SY5Y cells and primary neurons) to glutamate excitotoxicity and oxidative stress in the presence and absence of PFT-α and a close analog (selected to illustrate that p53 inhibition-associated neuroprotection was not compound specific). This was undertaken as oxidative stress and overwhelming disturbances in cellular ion homeostasis, particularly calcium ions, have been reported to occur following TBI that are activated by the excessive release of excitatory amino acids, chiefly glutamate, triggering the subsequent stimulation of glutamate receptors [39,75]. Calcium ion cellular influx is a key incident early post-TBI and provokes mitochondrial damage with an uncoupling of mitochondrial ATP synthesis, a rise in free radical production, alterations in gene expression, the activation of calcium-dependent proteases inducing cellular damage, and a critical rise in p53 levels to instigate apoptosis [14]. Elevated extracellular glutamate levels are hence believed to be key in mediating primary and secondary damage in mTBI as well as in cerebral ischemia [76,77] and many neurodegenerative diseases [78]. Glutamate excitotoxcity and oxidative stress dramatically elevates p53 levels in neurons [29], and PFT-α effectively blocked cell death (Figure **2**) in accord with results from others as well as in response to other apoptosis-inducing insults [29,74,79]. Likewise, the natural time-dependent degeneration of primary neuron [44] was mitigated by PFT-α.

The inhibition of p53 activity, the principle action of PFT-α and analogs to prevent the pro-apoptotic action of this tumor suppressor protein, may involve inhibition of p53 accumulation, inhibition of translocation from cytoplasm to the nucleus or mitochondria, or interference of the p53 transcriptional machinery, or a combination by PFT-α [80,81]. In general, p53 activity is chiefly determined at its protein level and, under normal circumstances, it has a short half-life and is hardly detectable in neurons. The interaction of p53 with the ubiquitin ligase, Mdm2, targets p53 for degradation by proteasomes. However in response to cellular stress, p53 becomes phosphorylated at specific serine and threonine residues and acetylated at several lysines. Such p53 phosphorylation reduces Mdm2 binding to support protein stabilization and a rise in intracellular p53 protein levels, and the acetylation increases p53 sequence specific DNA binding to facilitate p53-mediated transactivation [82]. The pro-apoptotic functions of p53 are largely promoted by effectors that include p53 upregulated modulator of apoptosis (PUMA) and Bax, both proapoptotic members of the Bcl-2 family. Additionally, p53 has the potential to induce cell death via transcriptional independent means, inhibiting endogenous survival cascades following its translocation to the mitochondria [81-83] or by processes involving transcriptional repression through interaction with transcriptional co-activating proteins (e.g., CBP/p300) to suppress the activity of transcription factors like nuclear factor-κB (NF-κB) [81,84]. 

In synopsis, p53 acts as a gateway to apoptosis in neurons following time-dependent degeneration [44] or an insult, whether glutamate excitotoxicity, oxidative stress, hypoxia, amyloid-β peptide or a toxin [80,81], and PFT-α and other potential drugs act as gatekeepers to fine tune the balance between cell death versus survival that can occur following mTBI, as in the current study, as well as after ischemic events and in neuropathological conditions [80,81,85]. The initiated time-dependent cascades can additionally be repressed, such as by elevating competing anti-apoptotic Bcl-2 family members as occurs with the administration of the glucagon-like peptide-1 agonist, exendin-4 [10,12,86], or by the use of Nrf2 activators [65,87]. PFT-α and other drugs are thereby useful as pharmacological tools to understand mechanisms underpinning the neuronal death and cognitive impairments induced by TBI (both moderate [23], and as shown herein, mild forms) as well as candidate drugs to alleviate these in an area of medicine in which no effective pharmaceutical therapy currently exists.
